# Studying functional networks in human brain through intracerebral spontaneous EEG

**DOI:** 10.1186/1471-2202-12-S1-P283

**Published:** 2011-07-18

**Authors:** Gabriele Arnulfo, Andrea Pigorini, Marcello Massimini, Lino Nobili, Andrea Schenone

**Affiliations:** 1 BioLab, Department of Communication, Computer and System Sciences (DIST), University of Genoa, Italy; 2 Department of Clinical Sciences, University of Milan, Milan, 20100, Italy; 3 Centre of Epilepsy Surgery "C.Munari", Department of Neuroscience, Niguarda Hospital, Milan, 20100, Italy

## 

Several experimental works have demonstrated that, apparently, spontaneous brain activity is not random. At the level of large-scale neural systems, the ongoing activity measured with functional MRI (fMRI) reflects the organization of a series of highly coherent functional networks [1]. Although methodologies based on fMRI are highly reliable in spatial resolution, they lack time resolution. To maximize the temporal resolution it is necessary to employ EEG-based methodologies. However, using EEG for studying functional connectivity is severely limited by volume conduction and by problems related to source modelling. To avoid these problems we propose an approach based on intra-cerebral EEG recordings (stereo-EEG) in human. Moreover, we measure functional connectivity applying mutual-information (MI) technique directly on segments of resting state stereo-EEG activity.

## Methods

Six patients with drug resistant epilepsy had been implanted with multi-channels depth electrodes for clinical evaluation [2]. The collected data are resting state, eye closed registrations divided into 30 time windows of 4 seconds each overlapped by 20% of their length. On these data we calculated the well established mutual information measure (MI) in order to individuate non-linear dependencies over time [3]. So far, for each bipolar signal and inside the same time window, we estimate the MI using the calculation of differential entropy [4]. The entire process outputs a functional square matrix where each position i,j contains the MI between bipolar source i against j. This matrix is normalized over its global maximum discarding self connections. Moreover, since we are mainly interested on measuring the functional distances to be able to compare them with geometrical distances, we computed a specific metric according the normalized mutual information matrix. Statistical analysis has been employed using Rank Order test, with p<0.05.

Moreover, we compute classical measures from graph theory in order to identify salient features from the network. The connection density of the network and the degree of each nodes have been calculated using BCT toolbox [5]. Since MI is a symmetric inter-dependence measure it cannot estimate the directionality of the connection, thus the resulting adjacency matrix will represent a undirected weighted graph. As a first approximation the binary threshold over the resulting weighted network has been calculated plotting the connection density over the NMI values and searching for the flex point in the resulting function.

## Results

Preliminary results over a single patient showed that a combination of stereo-EEG and MI is a useful tool to study functional networks in human brain. In particular, the functional network obtained by calculating MI among stereo-EEG recordings is scale-free since its connection density declines with a power low according to P(k) = ck-2. With such a method we could easily individuate small clusters of brain regions sharing information and those who could be supposed to be hubs or able to trigger cerebral events.
